# How psychosocial factors affect well-being of practice assistants at work in general medical care? – a questionnaire survey

**DOI:** 10.1186/s12875-015-0366-y

**Published:** 2015-11-11

**Authors:** Katja Goetz, Sarah Berger, Amina Gavartina, Stavria Zaroti, Joachim Szecsenyi

**Affiliations:** Department of General Practice and Health Services Research, University Hospital of Heidelberg, Vosstr. 2, Building 37, Heidelberg, D – 69115 Germany; Institute of Family Medicine, University Hospital Schleswig-Holstein, Campus Luebeck, Ratzeburger Allee 160, Luebeck, D – 23538 Germany

**Keywords:** General medical practices, Practice assistants, Psychosocial factors, Workforce strategy

## Abstract

**Background:**

Well-being at work is an important aspect of a workforce strategy. The aim of the study was to explore and evaluate psychosocial factors and health and work-related outcomes of practices assistants depending on their employment status in general medical practices.

**Methods:**

This observational study was based on a questionnaire survey to evaluate psychosocial aspects at work in general medical practices. A standardized questionnaire was used, the Copenhagen Psychosocial Questionnaire (COPSOQ). Beside descriptive analyses linear regression analyses were performed for each health and work-related outcome scale of the COPSOQ.

**Results:**

586 practice assistants out of 794 respondents (73.8 %) from 234 general medical practices completed the questionnaire. Practice assistants reported the highest scores for the psychosocial factor ‘sense of community’ (mean = 85.9) and the lower score for ‘influence at work’ (mean = 41.2). Moreover, practice assistants who worked part-time rated their psychosocial factors at work and health-related outcomes more positively than full-time employees. Furthermore, the two scales of health related outcomes ‘burnout’ and ‘job satisfaction’ showed strong associations between different psychosocial factors and socio-demographic variables.

**Conclusions:**

Psychosocial factors at work influence well-being at work and could be strong risk factors for poor health and work-related outcomes. Effective management of these issues could have an impact on the retention and recruitment of health care staff.

## Background

Sense of well-being at work is an important aspect of a workforce strategy. In recent years, close attention has been paid to this especially for health care professionals. In western nations, unattractive working conditions in combination with work-related stress, an aging population and workforce shortages all highlight the need for up-to-date research in this field. Well-being can be described as a summative concept and includes “physical, emotional and social factors, both inside and outside the workplace” [1] and is a major determinant of productivity [2]. An important impact on well-being at work can be attributed to psychosocial work environment including work climate, work recognition, and social support [3]. Psychosocial factors comprise aspects such as workplace social support, job satisfaction or physical load at work [4].

In recent years, different work-related factors impacting on well-being at work for have been evaluated. For example, there was a reported positive association between medication error, working overtime and poor job security for nurses [5]. It has also been shown that higher qualified health care professionals resulted in a lower mortality rate for patients [6]. Moreover, working conditions also impact on mental and physical health of employees. Ganster and Rosen reported that workplace demands could produce different changes in mental and physical health [7]. From the job demands-control model, it is known that there is a relationship between stress factors at work and the impact on health complaints [8]. For health care professionals, it has been shown that stress-related working conditions, such as a high proportion of working hours, shift work and a high numbers of patient contacts could result in emotional exhaustion, psychosomatic health complaints and a higher turnover rate [9–11].

Most of these studies focused on health care professionals in the hospital setting. Little is known about psychosocial factors at work for practice assistants in general medical practice and impacts on health and work-related outcome such as burnout, general health or stress symptoms. Nevertheless, it should be a research topic with high priority for future health care research, in particular due to workforce shortages and challenges in recruitment and retention of staff in general medical practices. Currently, there are 398,000 practice assistants working in ambulatory care settings (e.g. general practice, out-patient services etc.) in Germany [12]. Of these, 100,700 practice assistants are employed by general practitioners, this equates to two or three per general practice [13]. There are no calculations at this stage in Germany for a recommended ratio of practice assistants to enrolled patient population for general practices. Practice assistants in Germany assist doctors with medical tasks, practice organization and administration. This is a recognized health workforce group *(Medizinische Fachangestellte)* with a three year part-time education programme at a vocational school [14]. They play an important role in assisting physicians in their daily activities to ensure quality patient care. Research into attractive working conditions and well-being at work for this workforce group can contribute evidence to support in the development of workforce strategies for their recruitment and retainment given current shortages in general practice services in Germany. The aim of the study was twofold: Firstly, we evaluated the psychosocial factors and health and work-related outcomes at work of practices assistants in general medical practices regarding their employment status. Secondly, we explored associations between psychosocial factors, and health and work-related outcomes of practice assistants to provide implications for further interventions in the general medical care sector.

## Methods

This observational study was based on a survey of psychosocial aspects at work in general medical practices in the German federal state of Baden-Wuerttemberg.

### Participants

The register of the National Association of Statutory Health Insurance Physicians of Baden-Wuerttemberg was used for the survey. For the selection of study population, simple random sampling was used. From 7319 registered general medical practices, 2000 were randomly selected for the study, 234 took part in this survey of practice assistants. These general medical practices received an invitation letter for the study which would survey their practice assistants. From these 234 general medical practices, 794 practice assistants were interested taking part in this study. Practice assistants were asked to fill in an anonymous paper-based questionnaire and to send it back to the study centre. The return of the anonymous paper-based questionnaire was classified as informed consent. We provided a free-post envelope, but no further financial incentives were offered for the participants and no reminder was sent out. Because this was an explorative study, no power calculation was determined.

### Psychosocial measures

Data collection took place between June and August 2011. Socio-demographic characteristics were included in the questionnaire, age as continuous variable and employment status as categorical variable, 35 hours and more (full-time) and 34 h or less (part-time). Besides the evaluation of socio-demographic characteristics of practice assistants, the questionnaire contained the German validated version of the Copenhagen Psychosocial Questionnaire (COPSOQ). COPSOQ is a well-established questionnaire and has been translated into more than 25 languages. The instrument could be used for the evaluation of psychological stress and strain at work and combines different measurements of psychological stress, work load and strain [15]. The COPSOQ questionnaire evaluates different psychosocial factors at work and health and work related outcomes. It was developed as a tool for assessment and improvement of the psychosocial work environment [16]. It consists of 25 scales which are classified to five thematic domains: “demands”, “influence and development”, “interpersonal relations and leadership”, “future parameters”, and “outcome scales” [17].

All items were assigned to these 25 scales. The number of items per scale is presented in Table 2. Each item is rated on a 4-point, 5-point or 7-point Likert scale and were transformed on a value range from 0 (minimum value) to 100 points (maximum value). For more information about the assignment of items to scales a download for the English version of the questionnaire is available on http://www.copsoq.de/copsoq-english. No adaption was made for this study.

### Statistical analysis

The analysis was performed with SPSS version 20.0 (SPSS Inc., IBM). Firstly, a descriptive analysis was undertaken to determine characteristics of the study population. Furthermore, descriptive analyses of the COPSOQ scales –different psychosocial factors at work and health and work related outcomes - were conducted. The means and standard deviations of theses scales are reported. Furthermore, non-parametric Mann–Whitney U test with listwise exclusion of missing data were used for group comparison between full-time/ part-time employees and the COPSOQ scales. Bonferroni correction was used for multiple testing. Afterwards, multivariate stepwise regression analyses were carried out. Each of the health and work-related outcomes was treated as a dependent variable and individual and practice characteristics and psychosocial factors at work were used as the independent variables. An alpha level of *p* < 0.05 was used for tests of statistical significance.

### Ethical approval

The study was fully approved by the ethics committees of the Medical Faculty of the University of Heidelberg (S137/2011). No additional data were evaluated.

## Results

794 practice assistants requested the questionnaire and a total of 586 practice assistants returned the questionnaire (73.8 % response rate). Data from non-responder were not available. The mean age of practice assistants was 39 years. Over 44 % of the practice assistants held a full-time position. More details of the characteristics of the study population are shown in Table 1.

**Table 1:** Characteristics of the study population

	Practices (*n* = 234)
Type of practice: solo handed	40.3 %
Location: urban	30.6 %
	Practice assistants (*n* = 586)
Age [mean (SD)]	39.1 (12.0)
Sex^a^ (female)	583 (99.5 %)
Employment status^a^	
Full-time worker	258 (44.0 %)
Part-time worker	322 (54.9 %)

*SD* standard deviation, ^a^n various due to missing data

### Evaluation of psychosocial factors at work and of health and work-related outcome

Table 2 presents the mean values and standard deviation of the different COPSOQ scales. Practice assistants reported the highest scores for the psychosocial factors ‘sense of community’ which means cooperation between colleagues (mean = 85.9), for ‘meaning of work’ which means motivated and involved in the work (mean = 83.8), and for ‘role-clarity’ (mean = 81.6). Furthermore, they reported lower scores for ‘degree of freedom at work’ (mean = 42.7), for ‘social relations’ (mean = 42.3), and for ‘influence at work’ (mean = 41.2). Regarding health and work-related outcomes, there were high scores for ‘general health’ (mean = 78.5), ‘job satisfaction’ (mean = 73.6), and ‘satisfaction with life’ (mean = 70.7).

**Table 2:** Psychosocial factors and health and work-related outcomes at work

COPSOQ scales^a^	Number of items	Mean	SD	95 % CI
Domain: Demands				
Quantitative demands	4	49.2	16.8	47.83–50.55
Emotional demands	3	47.9	19.2	46.32–49.43
Demands for hiding emotions	2	44.9	23.1	43.01–46.75
Work-Privacy-Conflict	5	25.4	24.4	23.43–27.39
Domain: Influence and development				
Influence at work	4	41.2	21.4	39.42–42.90
Degree of freedom at work	4	42.7	20.0	41.05–44.30
Possibilities for development	4	69.1	14.5	67.87–70.22
Meaning of work	3	83.8	15.2	82.52–84.99
Workplace commitment	4	64.0	18.0	62.46–65.31
Domain: Interpersonal relations and leadership				
Predictability	2	67.0	21.0	65.34–68.72
Role-clarity	4	81.6	13.7	80.43–82.66
Role-conflict	4	29.2	21.0	27.48–30.90
Quality of leadership	4	65.9	20.9	64.13–67.57
Social support	4	78.2	18.5	76.73–79.74
Feedback	2	51.4	23.1	49.52–53.27
Social relations	2	42.3	16.9	40.92–43.66
Sense of community	3	85.9	15.9	84.63–87.22
Workplace bullying (single item)	1	17.6	22.2	15.83–19.45
Domain: Further parameters				
Job insecurity	4	20.2	18.0	18.74–21.66
Domain: Outcome scales				
Thinking about early retirement (single item)	1	15.4	23.1	13.45–17.24
Job satisfaction	7	73.6	14.0	72.47–74.76
General health	1	78.5	18.8	76.54–79.61
Burnout	6	38.0	19.8	36.41–39.63
Cognitive stress symptoms	4	26.4	18.2	24.88–27.84
Satisfaction with life	5	70.7	18.1	69.20–72.15

*SD* standard deviation, 95 % *CI* 95 % confidence interval

^a^Possible score for each scale between 0 (minimum) and 100 (maximum)

### Comparing of full-time and part-time employees regarding COPSOQ scales

Table 3 shows the comparison of part-time and full-time employees regarding COPSOQ-scales. Practice assistants who worked full-time reported significantly higher scores with ‘quantitative demands’, ‘emotional demands’, ‘work-privacy-conflict’, ‘influence at work’, ‘possibilities for development’, and ‘burnout’ than practice assistants who worked part-time.

**Table 3:** Psychosocial factors at work and work-related outcomes compared to full-time and part-time employment

COPSOQ scales^a^	Full-time Mean (*n* = 241)	Part-time Mean (*n* = 295)	p-value
Domain: Demands			
Quantitative demands	52.6	47.0	<0.01*
Emotional demands	50.8	46.6	0.005
Demands for hiding emotions	44.8	46.4	0.547
Work-Privacy-Conflict	35.7	18.0	<0.01*
Domain: Influence and development			
Influence at work	42.5	39.1	0.043
Degree of freedom at work	42.5	42.6	0.889
Possibilities for development	70.4	68.1	0.035
Meaning of work	84.3	83.2	0.343
Workplace commitment	64.8	63.5	0.332
Domain: Interpersonal relations and leadership			
Predictability	65.8	68.0	0.076
Role-clarity	81.0	81.8	0.631
Role-conflict	34.9	24.1	<0.01*
Quality of leadership	63.8	68.0	0.011
Social support	77.8	78.8	0.326
Feedback	52.0	51.2	0.670
Social relations	40.8	42.9	0.155
Sense of community	85.9	86.3	0.779
Workplace bullying (single item)	20.5	14.6	0.025*
Domain: Further parameters			
Job insecurity	21.3	19.3	0.133
Domain: Outcome scales			
Thinking about early retirement (single item)	20.0	12.3	<0.01*
Job satisfaction	71.9	75.1	0.003
General health	76.7	78.7	0.408
Burnout	44.3	33.5	<0.01*
Cognitive stress symptoms	28.5	25.0	0.037
Satisfaction with life	67.0	73.6	<0.01*

^a^Possible score for each scale between 0 (minimum) and 100 (maximum)

*Bonferroni correction, statistical significance *p* < 0.05

### Factors associated with health and work-related outcomes

The associations between individual and practice characteristics and psychosocial factors at work concerning health and work-related outcomes are presented in Table 4.

**Table 4:** Impact of psychosocial factors on health and work-related outcomes (results of the forward stepwise regression analyses)

Outcomes	Scales	Beta	R^2^
Burnout	Cognitive stress symptoms	0.33	0.67
	Work-Privacy-Conflict	0.22	
	General health	−0.29	
	Age of practice assistants	−0.14	
	Emotional demands	0.17	
	Satisfaction with life	−0.09	
	Role-conflict	0.07	
	Demands for hiding emotions	−0.06	
Job satisfaction	Quality of leadership	0.28	0.60
	Sense of community	0.20	
	Workplace commitment	0.17	
	Role-clarity	0.11	
	Satisfaction with life	0.10	
	Quantitative demands	−0.10	
	Meaning of work	0.11	
	Cognitive stress symptoms	−0.07	
	Role-conflict	−0.06	
	Demands for hiding emotions	−0.08	
	Age of practice assistants	0.06	
General health	Burnout	−0.54	0.39
	Age of practice assistants	−0.14	
	Job satisfaction	0.09	
	Influence at work	0.09	
	Satisfaction with life	0.08	
	Work activity	−0.07	
Satisfaction with life	Burnout	−0.21	0.24
	Think about early retirement	−0.14	
	Job satisfaction	0.17	
	Employment status	0.14	
	General health	0.14	
	Predictability	−0.14	
	Influence at work	0.09	
Cognitive stress symptoms	Burnout	0.63	0.43
	Employment status	0.11	
	Job satisfaction	−0.11	
Thinking about early retirement	Job satisfaction	−0.13	0.28
	Burnout	0.15	
	Meaning of work	−0.13	
	Satisfaction with life	−0.14	
	Sense of community	−0.15	
	Influence at work	0.10	
	Work-Privacy-Conflict	0.12	

*R* ^*2*^ Proportion of the variance explained by the model

All coefficients are statistically significantly at the *p* < 0.05 level

Three of the six regression models explained more than 40 % (R^2^ > 0.40) of the variance of the dependent variables. These three variables were ‘burnout’, ‘job satisfaction’, and ‘cognitive stress symptoms’. The two outcome variables of health and work-related aspects that were best explained by the variance of the independent variables were ‘burnout’ (R^2^ = 0.67) and ‘job satisfaction’ (R^2^ = 0.60). Higher scores of ‘cognitive stress symptoms’, ‘work-privacy-conflict’, ‘emotional demand’, and ‘role-conflict’ and lower scores of ‘general health’, ‘satisfaction with life’ and ‘demanding for hiding emotions’ and younger practice assistants explained a higher risk of burnout. Furthermore, job satisfaction was associated with higher scores of ‘quality of leadership’, ‘sense of community’, ‘workplace commitment’, ‘role-clarity’, ‘satisfaction with life’, ‘meaning of work’, and lower scores of ‘quantitative demands’, ‘cognitive stress symptoms’, ‘role conflict’, ‘demanding for hiding emotions’ and older practice assistants.

## Discussion

To our knowledge, this was the first study that uses the COPSOQ questionnaire for practice assistants in a general medical care setting in Germany, where to date there has been little published research in this field. Our study contributes early evidence to a field with increasing importance due to issues with general practice workforce shortages in Germany. It was found that practice assistants showed high level of sense of community, meaning of work, and role-clarity which are crucial aspects for intrinsic motivation at work. Previous studies have reported that clear responsibilities and also extension of roles of health care staff resulted in a higher job satisfaction and identification with their job [18–20]. Moreover, job satisfaction is an important predictor of intention to stay in the job but could be influenced by organizational attributes within the practice [21, 22].

It was found that practice assistants who worked part-time showed lower levels of quantitative and emotional demands, work-privacy conflict, workplace bullying, thinking about early retirement and burnout. Furthermore, for this group higher job satisfaction and higher level of satisfaction with life were observed. In addition, the results demonstrated that practice assistants who worked part-time rated their psychosocial factors at work and health-related outcomes more positive than full-time employees. The same result was found in a German-wide study about job satisfaction surveying practice assistants where the part-time workers were more satisfied with their job than full-time staff [18]. Furthermore, a study with employees from the service sector in five western European countries demonstrated that working part-time positively affect work-family balance [23]. In our study and general, nearly 100 % of practice assistants in general medical practices are female, for whom the opportunity to work in a part-time position to juggle family and work is often desirable. Therefore, a prospective study followed by a targeted intervention would be necessary to consider employment status and working condition for recruitment and retention of practice assistants.

Furthermore, it has been demonstrated that different psychosocial factors at work are associated with health-related outcomes. The two scales of health related outcomes ‘burnout’ and ‘job satisfaction’ showed strong associations between different psychosocial factors and socio-demographic variables with an explained variance of 67 % resp. 60 %. These results compared favorably to a study with service personnel, which also used the COPSOQ questionnaire and showed that psychosocial factors at work strongly related to the risk of burnout (24). In our study, a number of psychosocial factors were associated with burnout, such as higher cognitive stress symptoms, work-privacy conflict, emotional demands, and role-conflict, lower general health, satisfaction with life, demanding for hiding emotions, and younger practice assistants. A study with primary care providers in the United States showed that physician assistants and nurse practitioners reported a higher level of stress than physicians [25]. A review by van Laar et al. showed that medical staff across different practice settings from 17 countries were stressed and strained [26].

It should be noted that a strong predictor for job satisfaction was the quality of leadership. A systematic review conducted in the hospital sector showed that leadership practices influences health care staff retention [27]. Moreover, it was found that authentic leadership positively influences health and retention of nurses and increased job satisfaction [28, 29]. It is to be recommended that general practitioners actively develop their own leadership skills depending on the individual workplace conditions, with the intent of enhancing their staff satisfaction and thereby, positively impacting on staff retention.

Furthermore, burnout and job satisfaction are strong predictors for the outcome item ‘thinking about early retirement’. It was shown that the relationship between work environment, working conditions and health are essential for retention of health care staff. Moreover, it was found that job satisfaction is highly associated with organizational attributes in primary care [22]. The establishment of healthy workplaces to positively impact on the recruitment and retention of staff in the health care sector cannot be underestimated [30]. Moreover, the work environment including psychosocial factors at work is an indirect predictor of quality of care, which should be addressed in further studies [3].

### Strengths and limitations

To our knowledge there is no published research for psychosocial factors at work of practice assistants in general medical practice in Germany. Our study provides an important contribution and provides early evidence in this field of research. The findings are tentative and it is not possible to determine cause-and-effect relationships. In addition, the use of an internationally validated instrument for the evaluation of psychosocial factors at work, the COPSOQ-instrument, enables international comparison and benchmarking of results [15–17]. Finally, our study had a good response rate from practice assistants in general medical practices (73.8 %). Data from non-responders were not evaluated. The non-response rate of 8.5 % was negligible. However, only limited conclusions can be drawn as we were unable to collect data on a broader range of socio-demographic characteristics including health risk behaviors of practice assistants. The participation of practice assistants in this study was voluntary. Therefore, a potential selection bias is indicated. In addition, as this was an exploratory study and the direction of the relationship cannot be determined, *p* values should be interpreted with caution. Significant results may be due to chance and will need to be confirmed in further targeted studies.

## Conclusions

Sense of well-being at work is dependent on multiple factors. With a view to the limitations of this explorative and cross-sectional study, our results could suggest that practice assistants who worked part-time rate their psychosocial factors at work and health-related outcomes more positively than full-time employees. Moreover, it was found that psychosocial factors at work are strong risk factors for poor health and work-related outcomes, especially those of burnout and lack of job satisfaction. Importantly, well-being at work was found to be related to quality of leadership. With the results of this study it can be assumed that improving leadership skills of general practitioners could positively impact on health and work-related outcomes, which in turn could positively influences the retention and recruitment of staff. However, there is a need for prospective studies followed by intervention to evaluate influencing factors.

## Abbreviations

COPSOQ: Copenhagen psychosocial questionnaire
SD: Standard deviation
95 % CI: 95 % confidence interval
R^2^: Proportion of the variance explained by the model
